# The influence of human milk composition and its microbiome on the gut microecology and early growth and development of preterm infants (the YI study): protocol design and cohort profile

**DOI:** 10.3389/fnut.2025.1566376

**Published:** 2025-07-30

**Authors:** Xinyue Wang, Yan Feng, Yilan Li, Yuluyuan Tian, Simin Zhang, Jinglin Li, Jiahui Zhang, Feng Liu, Jiahe Zhou, Ting Li, Sufang Duan, Ignatius Man-Yau Szeto, Li Su, Xiaoqin Luo

**Affiliations:** ^1^Department of Nutrition and Food Safety, School of Public Health, Xi'an Jiaotong University, Xi'an, China; ^2^School of Public Health, Institute of Maternal, Child and Adolescent Health, Lanzhou University, Lanzhou, China; ^3^Inner Mongolia Yili Industrial Group Co., Ltd., Hohhot, China; ^4^Inner Mongolia Dairy Technology Research Institute Co., Ltd., Hohhot, China; ^5^National Center of Technology Innovation for Dairy, Hohhot, China; ^6^Key Laboratory for Disease Prevention and Control and Health Promotion of Shaanxi Province, Xi'an, China

**Keywords:** preterm infants, human milk, gut microecology, early life health, systems biology

## Abstract

**Introduction:**

Breastfeeding can reduce the risk of serious illness in preterm infants. However, the influence of human milk on the gut microecology and early development of preterm infants remains unclear.

**Methods:**

The YI Study is a prospective cohort protocol conducted in China, designed to investigate the dynamic associations among breast milk composition, infant gut microecology, and health from a mother–breastmilk–preterm infant triad perspective. From January 2023 to May 2024, a total of 50 mother–term infant dyads and 35 mother–preterm infant dyads were enrolled and followed up at six timepoints: v1 (0–7 days), v2 (8–14 days), v3 (1 month), v4 (2 months), v5 (4 months), and v6 (6 months). Data collection included questionnaires, anthropometric measurements, and biospecimens. Questionnaires (including birth medical records, environment, feeding practices and illnesses status) and anthropometric measurements were collected at all visits. Biospecimens included paired samples of breast milk and feces were obtained at each visit, and comprehensively analyzed by multi-omics techniques. We also collected heel blood at birth to examine immune status and saliva at the v6 visit to explore the role of its constituents in dietary behaviors.

**Results:**

The average age of the mothers was 30.9 ± 3.5 years. The median gestational age was 36 (35, 36) weeks in the Preterm Group and 39 (38, 40) weeks in the Term group. The completion rate up to V6 was 82.9% in the Preterm Group and 94% in the Term Group. All samples were collected within the predefined visit windows, with a total of 452 breast milk, 465 infant feces, 227 maternal feces, 49 heel blood and 98 saliva.

**Discussion:**

Through ultra-early, multi-temporal, multi-sample collection, combined with multi-omics technologies, the YI study will provide an opportunity to explore the dynamic association of human milk as a complex biological system with gut microecology and health in preterm infants in depth.

## 1 Introduction

Rapid advances in medicine and intensive care technology in recent years have significantly improved the survival rate of preterm infants. However, the incidence of serious conditions in preterm infants remains notably high (1) and the factors affecting the growth and development of premature infants should be more widely explored.

Breastfeeding is essential to establish healthy gut microbiome (2) and reduce the risk of serious diseases (3–5). However, the breastfeeding rates among preterm infants in China remain low, with less than 30% being breastfed within 6 months of delivery (6–10). In addition, although breast milk is considered the “gold standard” of nutrition for healthy infants, it may not fully meet the specific nutritional needs of preterm infants (11). Premature birth may alter biological and hormonal signals, and prompt changes in breast milk composition and gut microbiome (12–14).

Existing cohorts of term infants have established extensive databases of breast milk (15–17). Increasing research has also been devoted to providing clues to the composition of breast milk and its association with the health of preterm infants. A meta-study systematically compared the essential fatty acid content of breastmilk in preterm infants across lactation periods with that of term mothers (18). A Spanish study found that Neuropilin-1 and kallikrein-6 in breast milk were higher in mothers with preterm infants and were associated with neural development (19). Additionally, the concentration of adipokines in breast milk of very preterm infants has been found to influence the length of infants, with MFG-E8 levels being lower in the breast milk of mothers whose infants developed delayed sepsis (4). Breast milk taken by preterm infants with necrotizing enterocolitis was found to have lower HMO concentrations, which also affected the development of gut microbiota in infants (20). Besides, feeding practices, mode of delivery and maternal diet have also been found to impact on the gut microbiome of preterm infants (21–23). However, simply analyzing the individual components of breast milk from preterm mothers is not sufficient to fully understand its complexity and its impact on health (24). Human milk needs to be studied as a biological system, especially for the special population of preterm infants. The physiological heterogeneity of the mother, the complex composition of the breast milk and the specific physiology of preterm infants are all part of a co-adaptive system, and changes in each system affect the development and health of the infant. Nevertheless, there is lack of preterm cohorts with a systems biology perspective to provide evidence for the specificities of the composition and microbiota of breast milk from preterm mothers and its impact on the gut microecology and health of preterm infants.

Therefore, this study aims to longitudinally investigate the associations within the mother-breastmilk-preterm infant triad, so as to provide a scientific basis for establishing a preterm infant breast milk database and promoting health research in preterm infants.

## 2 Methods and analysis

### 2.1 Study design

**Figure 1:**
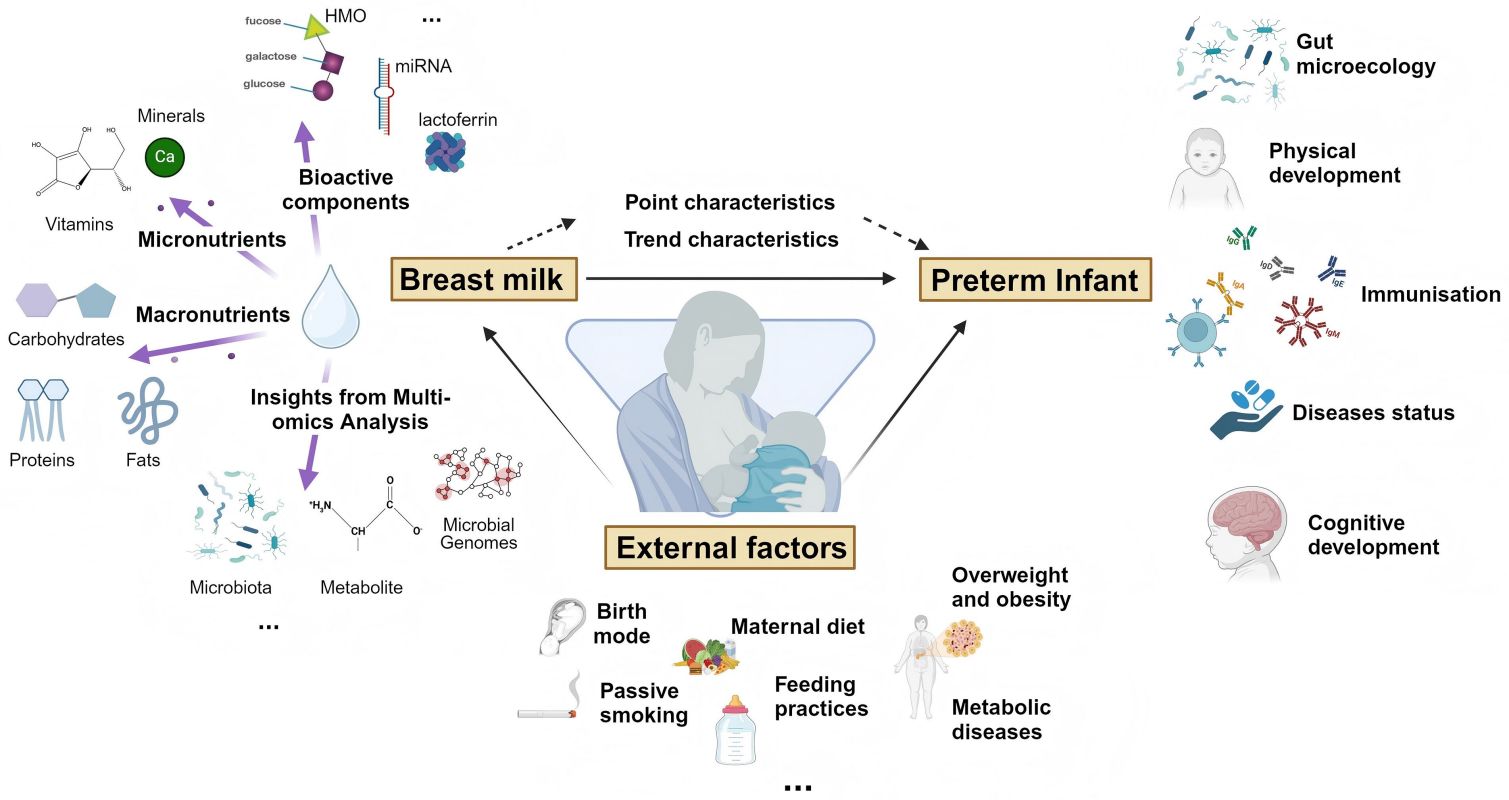
Visualization chart for YI study aims. Created with BioRender.com.

The YI study is a multicenter, prospective cohort study conducted in 4 tertiary hospitals in Xi’an and Lanzhou in China, including preterm and term mother-infant dyads, followed from birth to 6 months of age. The aims of the cohort study were (Figure 1):

1. To detect the content of nutrients and bioactive components of breast milk and their dynamic patterns, and explore their impact on the establishment of gut microbiota in preterm infants during the early stages of life;
2. To reveal the dynamic interactions between breast milk and gut microecology of preterm infants at multiple time points, and to identify the key factors of interactions through multi-omics techniques;
3. To study the immune status of preterm infants after birth by testing blood immune factors. To examine the health status, developmental level and disease risk of preterm infants by multi-omics analyses of saliva;
4. To study the effects of internal factors (immune status and gene) and external factors (birth mode, maternal diet, feeding practices, overweight and obesity, metabolic diseases, passive smoking and so on) on breast milk and infant gut microecology.

The study was approved by the Research Ethics Committee of Xi’an Jiaotong University (XJTU2022-1486) and registered with the Chinese Clinical Trial Registry (ChiCTR2200064473). All participants were informed of the study’s purpose, procedures, potential benefits and risks prior to enrollment, and written informed consents were obtained during the initial survey. This study was conducted in accordance with the principles in the Declaration of Helsinki.

### 2.2 Participant selection

Mother-infant dyads were recruited from the four centers for antenatal care and delivery. Mothers were screened about 4 weeks before the expected date of delivery and infants were screened within 7 days of delivery, with only mother-infant dyads both fitting the criteria being included in the study. Based on gestational age, dyads were divided into Preterm Group (born at 32–36^+6^ weeks of gestational age) and Term Group (born at 37–41 weeks of gestational age) (see Table 1).

**Table 1:** Inclusion and exclusion criteria.

Inclusion criteria	Exclusion criteria
Mothers: 18–40 years old; Planning breastfeeding or mixed feeding;	Mothers: With tumors, chronic diseases (e.g., Hypertension, diabetes, chronic hepatitis, chronic nephritis), autoimmune diseases or mental illnesses; Smoking or drinking alcohol during pregnancy; Using antibiotics or steroids within 30 days before delivery or taking regular probiotics; Participation in other studies within 2 weeks before study entry;
Infants: Single live birth; Breastfeeding or mixed breastfeeding at entry.	Infants: With congenital anomalies, genetic diseases or chromosomal disorders; With any disease requiring mechanical ventilation or medication (excluding jaundice in infancy treated with blue light) within one week of birth; birth through assisted reproductive technology (ART).

### 2.3 Follow up

**Figure 2:**
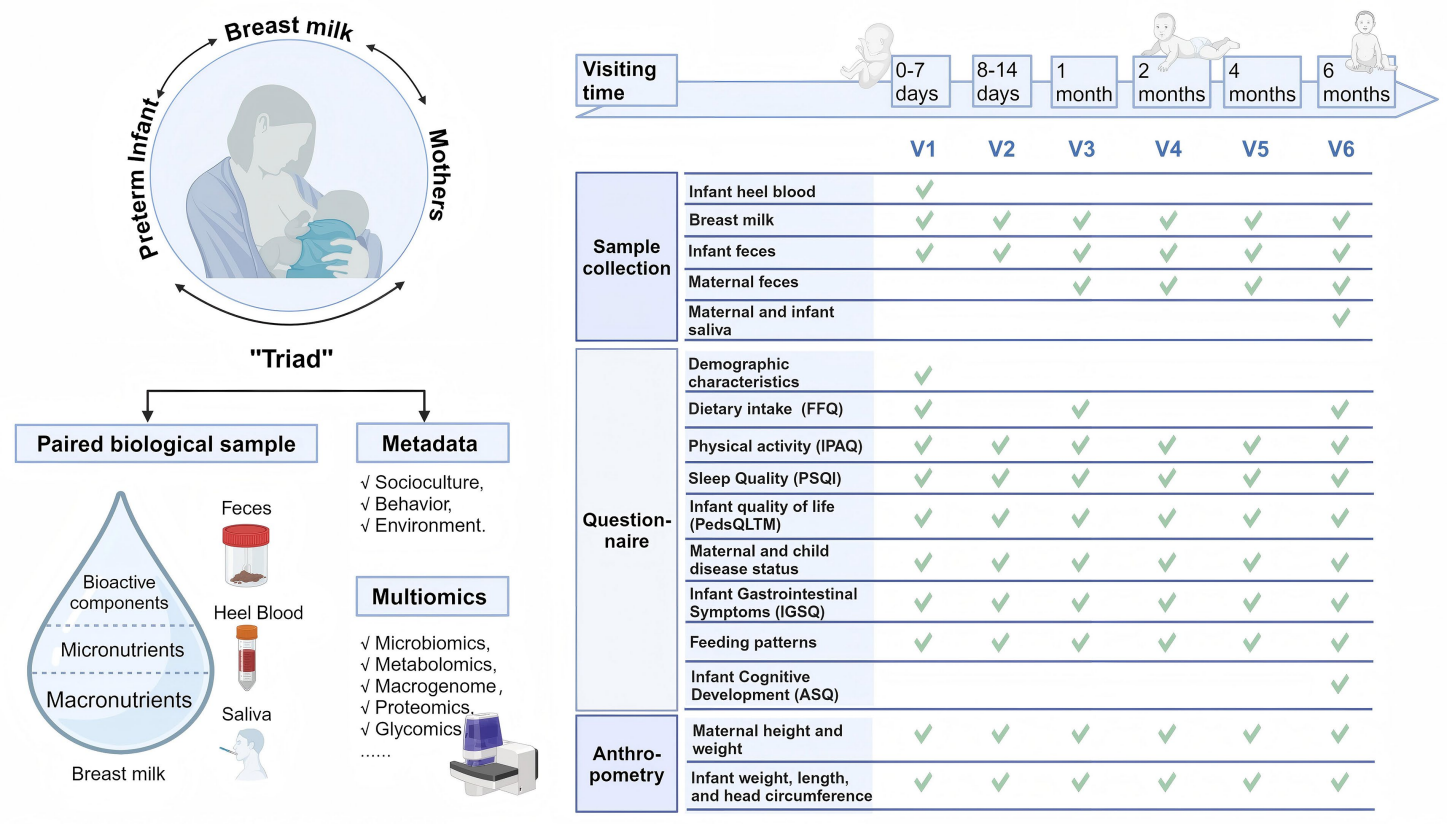
The principle for cohort establishment and time points for dyads data and sample collection. Created with BioRender.com.

All mother–infant dyads were followed up immediately after birth, with a total of six visits up to the six-month age of the infant: v1 (0–7 days), v2 (8–14 days), v3 (1 month±1 week), v4 (2 months±1 week), v5 (4 months±2 weeks), and v6 (6 months±2 weeks). The collection of biospecimens, questionnaires and anthropometric measurements was conducted following the theory of the mother-breastmilk-infant triad, as detailed in Figure 2.

### 2.4 Date collection

#### 2.4.1 Questionnaires

Information was collected at different time points using a set of validated questionnaires, which were administered through face-to-face interviews, as shown in Table 2.

**Table 2:** Questionnaire information.

Domain	Questionnaire content
General information	(1) Basic information: age, ethnicity, marital status, educational level, occupation, annual income of parents; (2) Maternity information: gravidity, parity, last menstrual date, expected date of delivery, pre-pregnancy BMI (weight and height), pre-pregnancy medical history, pregnancy complications and breastfeeding experience; (3) Birth information: date of birth, gestational age, gender, mode of delivery and time of first breastmilk intake; (4) Living environment: household size, housing area, energy usage in kitchen, pets and exposure of mothers and children to second/third-hand smoke.
Dietary intake	The Food Frequency Questionnaire (FFQ) (25) was used to recall the frequency and amount of maternal dietary intake in the past week/month, based on the food atlas (26). The FFQ consists of 13 major categories: grains and cereals, legumes, meat and fish, eggs, dairy, mushrooms and algae, vegetables, fruits, nuts, pastries, beverages, cooking oils and nutritional supplements.
Physical activity	The International Physical Activity Questionnaire (IPAQ) short version (9 items) (27) was used to assess the intensity and duration of the mother’s ≥ 10 min of activity and sedentary time in the past week. The intensity of activity was graded as walking, in moderate- and vigorous-intensity activity.
Sleep quality	The Pittsburgh Sleep Quality Index (PSQI) (28) was used to assess the quality and disturbance of mothers’ sleep over the past week. There are five “component” scores: sleep latency, sleep duration, sleep disturbance, daytime dysfunction and subjective sleep quality.
Feeding patterns	This section includes both feeding behaviors and feeding experiences. (1) Feeding behavior includes the infant’s feeding patterns over the past month, including five categories: direct breastfeeding, breastmilk bottle-feeding, infant formula, mixed feeding and complementary food addition. Additionally, the first time of breastfeeding, formula and complementary foods, their frequency and quantity of infant intake in the last week, mixed feeding practices, and reasons for stopping breastfeeding were also collected; (2) Feeding experiences investigated mothers’ actual experiences of breastfeeding (direct, bottle), planned breastfeeding duration, and reasons for and experiences with choosing formula feeding.
Pediatric quality of life	The Pediatric Quality of Life Inventory™ (PedsQL™) (29) can be used to assess the health-related quality of life in infant up to 12 months of age. The questionnaire includes five dimensions: physiological functioning, physical symptoms, emotional function, social function, and cognitive function.
Infant gastrointestinal symptom	The Infant Gastrointestinal Symptom Questionnaire (IGSQ) (30) was used to investigate the frequency, severity and amount of five gastrointestinal symptom clusters in infants over the past week. The 5 symptom clusters include stooling, spilling/vomiting, crying, irritability and flatulence.
Maternal and child disease status	Information on the types and severity of illnesses and the use of medication for mothers and infants since delivery or the last visit was collected through maternal self-report. For mothers, illnesses included mammary gland diseases, respiratory infections, gastrointestinal infections and other diseases. For infants, the reported illnesses included respiratory infections, gastrointestinal infections, skin rashes and other conditions.
Infant cognitive development	Ages & Stages Questionnaires, Social–Emotional Second Edition (ASQ:SE-2) (31) was used to assess the development of social–emotional behaviors in children aged 1–72 months (corrected age) across 7 functional domains: self-regulation, compliance, social-communication, adaptive function, autonomy, emotion and interpersonal interaction. The questionnaire results were collected through a web-based system (www.neoballoon.com) with automatic scoring. Cognitive function in infant subjects was classified into three levels: above-, near- and below-boundary on comparisons with the standard normal model of Chinese children (32).

#### 2.4.2 Anthropometry

All measurements were carried out by two trained investigators. The anthropometry of the mother included height and weight. The weight of the mothers was measured statically with a calibrated weighing scale (Yiqing, Senssun, Zhongshan, China) without shoes and heavy clothing and recorded with an accuracy of 0.1 kg. The height of the mother was recorded in centimeters to an accuracy of 0.1 cm.

Infancy anthropometry included length, weight and head circumference. Length and weight were measured by the electronic infant scale (iR-Baby, Senssun, Zhongshan, China) with an accuracy of 0.1 cm and 5 g, respectively. The scale was placed horizontally on the floor and the child was placed on the scale in a supine position for length and weight readings. Head circumference was measured using a soft tape (Wentai, Foshan, China), positioned around the head at the midpoints of the two eyebrow arches and the occipital tuberosity (the largest protuberance) with an accuracy of 0.1 cm.

#### 2.4.3 Biospecimens

Heel blood of infants at birth, breast milk (all visits), feces (all visits) and saliva (at the v6 visit) of mothers and infants were collected. All samples were transported at −20° after sampling and transported back to the central laboratory within 10 h for storage at −80°C.

##### 2.4.3.1 Heel blood

Under aseptic conditions, the clinical researchers placed the neonate in the supine position, and stabilized and engorged the infant’s plantar puncture site. After skin disinfection, a sterile needle was used to puncture the plantar foot swiftly in either a straight or oblique puncture. A drop of blood was deposited onto an FTA card, with a total volume of 2–3 mL. After the dried blood spot was made, placed the FTA card immediately into a sterile EP tube and stored at room temperature.

##### 2.4.3.2 Human milk

The collections were all conducted between 9 a.m. and 11 a.m. The sampled breast was required to be neither breastfed nor pumped for at least 2 h before sampling, and no skin care products, cosmetics, or other reagents were applied for 24 h before collection. The breast milk on the sampled side was required to be completely excreted and mixed thoroughly in a sterile breastmilk bottle, and then taken in volumes of 10 mL (0–14 days) or 40–50 mL (≥1 month) transferred to 1 mL, 2 mL, 5 mL, and 10 mL tubes, respectively.

##### 2.4.3.3 Infant and maternal fecal sample collection

Infant fecal samples were collected by scraping feces from the central part of the stool that had not touched the diaper with moderate hardness. Samples were stored in collection boxes filled to approximately 1/3 to 2/3 of their volume.

Maternal feces were self-collected by participants following standardized procedures. Prior to sampling, participants were instructed to urinate, flush the toilet, and place the plastic bidet-style stool collection box in a clean toilet seat. Participants used clean spoons to scrape feces from the middle and inside of feces that had not touched the toilet or external surfaces with moderate consistency. A volume equivalent to 1/3 to 2/3 of the collection cassette was retained.

Additionally, an extra portion of fecal samples was collected into microplastic-free storage tubes at each sampling event. Both infant and maternal fecal samples were collected using disposable stainless steel spoons and stored in stainless steel collection tubes. The scraping position and procedure adhered to the standard sample collection protocol.

##### 2.4.3.4 Infant and maternal saliva collection

Infant saliva was collected by researchers wearing disposable medical gloves when infants were emotionally stable and had not been fed for 2 h.

A sterile cotton swab was gently rubbed along the inside of the infant’s mouth for 2–3 min. Once at least 1/3 of the swab was saturated, store it in a collection tube with a tight-fitting lid.

Maternal saliva was self-collected by participants after fasting for at least 30 min and rinsing with water before collection. A sterile cotton swab was placed in the mouth for 2–3 min (without chewing) and stored in the collection tube with the lid closed after at least 1/3 of the swab was soaked.

### 2.5 Quality control

All investigators were trained in Good Clinical Practice (GCP) guidelines and were guided by a well-established program survey operations manual. All on-site processes of the questionnaires and samples were conducted with the assistance and supervision of the investigators. The collection and administration of questionnaires and biological samples for each subject were also the responsibility of the designated investigator (assigned by the center director).

Within 1 week after the end of the survey, all paper-based information was digitized using the research electronic data capture (REDCap (33)) tools at the School of Health, Xi’an Jiaotong University, and entered by the researcher in charge of the on-site survey. The REDCap system provides the first line of quality defense for the consistency, completeness, and logicality of the research data, and ensures data security. System permissions restricted investigators to access only the questionnaires they entered, whereas the center manager had access to all questionnaires. The center manager provides the second line of defense for the integrity and logic of the data. Logical corroboration was performed both within individual questionnaires and across different questionnaires for the same subject. Paper-based records (questionnaires and sampling information forms) were uniformly preserved by the center manager and sealed after verification and entry. All recorded information remained traceable to the original documents.

### 2.6 Laboratory test

Dried blood spot samples were analyzed using the OLINK Target 96 Inflammation panel, which analyzed the levels of 92 proteins in blood samples primarily associated with immune and inflammatory biological processes, based on proximity extension assay (PEA) technology.

Micronutrients in breast milk: vitamins were analyzed by liquid chromatography-mass spectrometry (LC–MS) and minerals by Inductively Coupled Plasma Mass Spectrometry (ICP-MS). Lactose in breast milk was analyzed by high-performance liquid chromatography with refractive index detection (HPLC-RID). Total protein was examined by Kjeldahl method; proteins were analyzed by LC–MS method targeting specific peptide fragments; and 16 amino acids were examined by automatic amino acid analysis (AAA), which is based on ion exchange chromatography coupled with ninhydrin chromogenic reaction. The lipids in breast milk, including fatty acids, phospholipids, glycolipids and structural lipids, were examined. Fatty acids were detected by gas chromatography with flame ionisation detection (GC-FID); phospholipids were measured by high-performance liquid chromatography with evaporative light scattering detection (HPLC-ELSD); glycolipids were tested by liquid chromatography–tandem mass spectrometry (LC–MS/MS); structural lipids were measured by silver ion normal-phase liquid chromatography with evaporative light scattering detection (Ag^+^-NP-LC-ELSD). The HMOs in breast milk were measured by liquid chromatography-high resolution mass spectrometry (LC-HRMS). Osmolality in breast milk was analyzed using freezing point osmometry (FPO).

The collected breast milk, fecal and saliva samples were analyzed in a multi-omics approach. 16S rRNA gene sequencing involves amplifying the hypervariable V3-V4 region of the 16S rRNA gene using polymerase chain reaction (PCR) and sequencing the amplicons with high-throughput sequencing platforms, to detect the diversity, abundance and taxonomic composition of microbiota. Metagenomic analysis involves random fragmentation of all microbial genomic DNA in the sample, followed by sequencing using high-throughput platforms, to analyze composition, functional genes and metabolic potential of the microbial community. Using untargeted metabolomics, LC–MS/MS was employed to analyze polar and non-polar metabolites, while gas chromatography-tandem mass spectrometry (GC–MS) was used for volatile and low molecular weight metabolites, to discover potential biomarkers and elucidates metabolic pathway alterations.

## 3 Result

**Figure 3:**
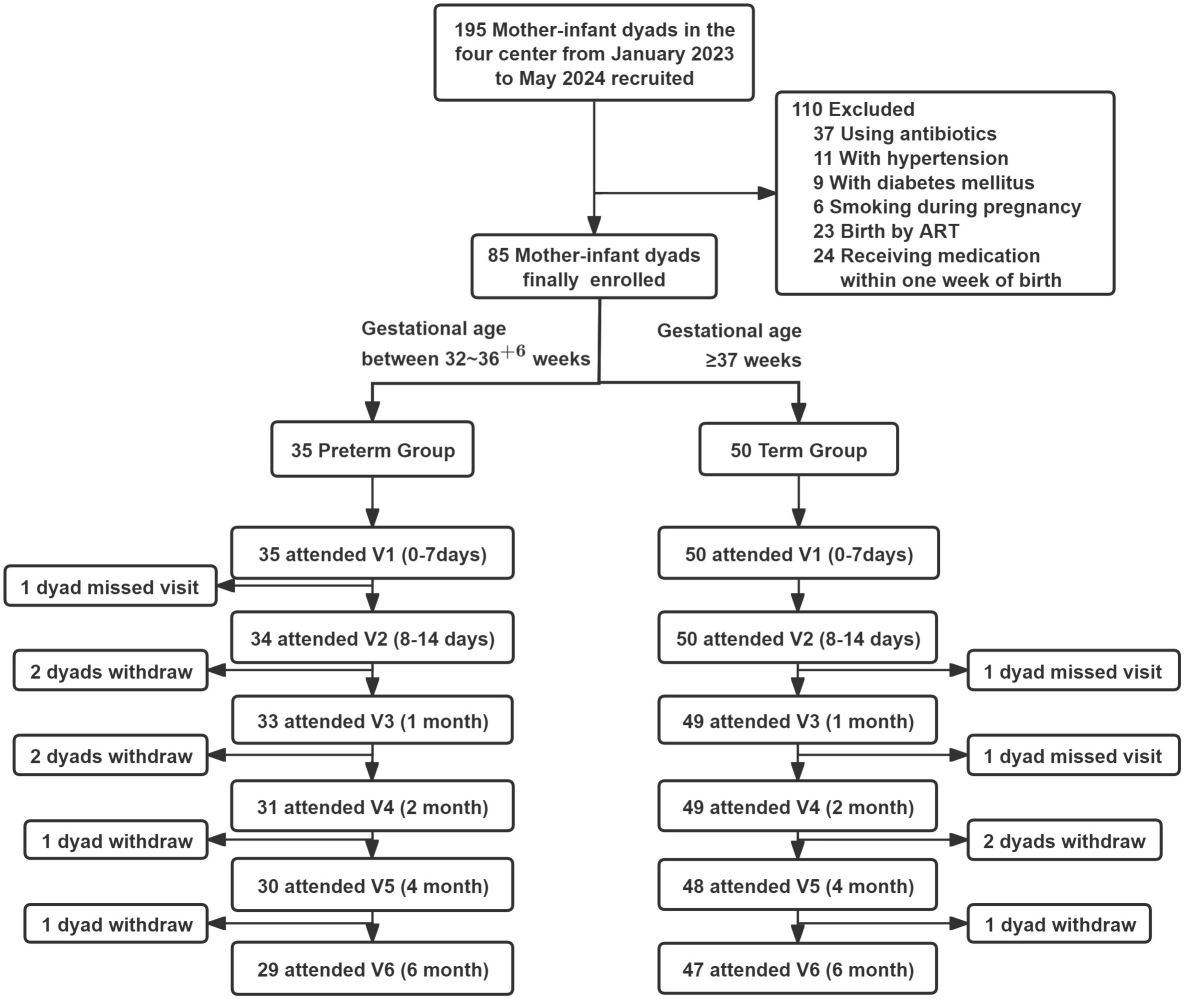
Flow chart of participants missed visit, defined as a follow-up visit that was skipped due to the participants left the place of the follow-up visit, but still attended the subsequent follow-up visits. Withdraw, means that participants completely quit the study.

From January 2023 to May 2024, a total of 50 mother-infant dyads of term infants and 35 mother-infant dyads of preterm infants were finally enrolled according to the inclusion and exclusion criteria (Figure 3). In the Preterm Infant Group, one mother was unable to attend the v2 visit due to work obligations; six mother-infant dyads dropped out between v3 and v6, giving a completion rate of 82.9% at v6. In the Term Infant Group, the completion rate remained 100% until v3, after which three mother-infant dyads withdrew, with a final completion rate of 94% at v6. All samples were collected within the predefined visit windows, with a total of 452 breast milk samples, 465 infant fecal samples, 227 maternal fecal samples, 49 heel blood samples and 98 saliva samples.

Baseline characteristics of mothers and infants in the cohort are presented in Table 3. The average age of the mothers was 30.9 ± 3.5. In the Preterm Group, the proportions of advanced maternal age (20%), multiparous women (22.9%), pregnancy complications (42.9%), and passive smoking exposure in both infants and mothers were higher than those in the Term Group. The median gestational age was 36 (35, 36) weeks in the Preterm Group and 39 (38, 40) weeks in the Term group. In the Preterm Group, 60% were delivered by cesarean section and 45.7% were small for gestational age (SGA).

**Table 3:** Baseline characteristics of mothers and infants in the study.

Characteristics	Total (*N* = 85)	Preterm Group (*N* = 35)	Term Group (*N* = 50)
Maternal characteristics			
Maternal age (years)	30.9 ± 3.5	31.5 ± 3.4	30.5 ± 3.5
<35 (%)	73 (85.9%)	28 (80.0%)	45 (90.0%)
≥35 (%)	12 (14.1%)	7 (20.0%)	5 (10.0%)
Maternal education (%)			
Low	7 (8.2%)	3 (8.6%)	4 (8.0%)
Middle	70 (82.4%)	29 (82.8%)	41 (82.0%)
High	8 (9.4%)	3 (8.6%)	5 (10.0%)
Family annually income (Ten Thousand yuan)			
≤15	55 (64.7%)	24 (68.6%)	31 (62.0%)
15–30	26 (30.6%)	9 (25.7%)	17 (34.0%)
≥30	4 (4.7%)	2 (5.7%)	2 (4.0%)
Parity (%)			
Primiparous	75 (88.0%)	27 (77.1%)	48 (96.0%)
Multiparous	10 (11.8%)	8 (22.9%)	2 (4.0%)
Maternal height (cm)	162.7 ± 5.4	162.7 ± 5.4	162.7 ± 5.4
Pre-pregnancy weight (kg)	57.5 (54.0, 65.0)	57.3 (53.7, 64.7)	57.5 (54.0, 65.0)
Pre-pregnancy BMI (kg/m^2^)	22.0 (19.8, 24.0)	22.6 (19.1, 24.1)	21.8 (19.9, 24.1)
Overweight (%)	19 (22.3%)	8 (22.9%)	11 (22.0%)
Obese (%)	4 (4.7%)	2 (5.7%)	2 (4.0%)
Postpartum weight retention at 6 months (kg)	4.1 ± 5.0	4.0 ± 5.0	4.1 ± 5.1
Pregnancy complications (%)	26 (30.6%)	15 (42.9%)	11 (22.0%)
Gestational diabetes mellitus	9 (10.6%)	4 (11.4%)	5 (10.0%)
Gestational hypertension	6 (7.1%)	5 (14.3%)	1 (2.0%)
Gestational hypothyroidism	7 (9.4%)	2 (5.7)	5 (10.0%)
Maternal exposure to passive smoke (%)	47 (55.3%)	22 (62.9%)	25 (50.0%)
Infant exposure to passive smoke (%)	39 (45.9%)	19 (54.3%)	20 (40.0%)
Infant characteristics			
Gestational age	37 (36, 39)	36 (35, 36)	39 (38, 40)
Caesarean section	44 (51.8%)	21 (60.0%)	23 (46.0%)
Birthweight (kg)	3.0 ± 0.6	2.5 ± 0.4	3.39 ± 0.4
SGA (%)	16 (18.8%)	16 (45.7%)	0 (0%)
Newborn length (cm)	49 (45, 48)	46 (45, 48)	50 (49, 50)
Newborn head circumference (cm)	34.0 (32.3, 36.9)	34.0 (32.7, 36.0)	34.5 (26.5, 37.0)

Education Level, Low: High school and below; Medium: University and college degrees; High: graduate school or higher. Maternal and Infant Exposure to Passive Smoke: Secondhand and thirdhand smoke exposure for mothers and infants. SGA: Small for gestational age.

## 4 Discussion

To the best of our knowledge, the YI study is the first prospective multimodal cohort study dedicated to investigating the associations between the complex components of breastmilk, gut microecology, and the health of preterm infants within the systems biology framework of the mother-breastmilk-preterm infant triad. By integrating data from multiple levels and time points, the YI study aims to comprehensively elucidate the critical role of breast milk in establishing gut microecology and promoting the health of preterm infants during the early stages of life, which provides the possibility of developing intervention strategies for this vulnerable population.

Breast milk establishes an efficient pathway for delivering nutrients and biologically active components, facilitating the vertical transmission of specific microbiome to infants (34–36). Importantly, breastmilk is not an isolated entity; its components are influenced by internal factors (lactating parent) and external factors (socioeconomic, cultural, behavioral and environmental contexts), all embedded within the mother-breastmilk-infant triad (37). From a systems biology perspective, the YI study cohort collected comprehensive metadata at multiple time points encompassing maternal demographic characteristics, environmental exposures, lifestyle factors (diet, sleep, exercise) and feeding practices. Paired biological samples were also systematically collected and the growth, development and health of infants have been tracked. By combining genomics, metabolomics, proteomics, microbiomics and other multi-omics technologies, multidimensional explorations of the complex interactions between breast milk and infant gut microecology can be realized in this cohort. The study design of YI study allows investigation of the effects of external factors on the triad, and the identification of bioactive breastmilk components that regulate preterm infant health. Notably, several critical areas remain underexplored in preterm infants, such as the association between bioactive components or microbiota in breast milk and the establishment of gut microecology, as well as the link between maternal nutrition and physiological status with breast milk characteristics and infant growth phenotypes, which could all be explored under this study design. The integration of diverse data types holds the potential to overcome the limitations of traditional research.

In addition, comprehensive and longitudinal data were incorporated in the study through ultra-early and multi-temporal follow-ups, covering colostrum, transitional milk, and various stages of maturation. Colostrum is the first colonizer of the infant intestine (34) and contains high concentrations of HMOs, secretory immunoglobulin A (IgA), lactoferrin, lysozyme and microRNA (miRNA) (38–40), which play critical roles in supporting gut microecology and immune system development (41–44). Compared to colostrum from term infants, that from preterm mothers has been reported to contain higher levels of immune components to address physiological immaturity (45, 46); however, these findings remain inconsistent (47) and lack evidence from Chinese populations. Studies have shown substantial microbial sharing between maternal breast milk and newborns, including the vertical transmission of *Bifidobacterium bifidum* (48). Nonetheless, these studies have focused on term infants and lacked systematic data on colostrum and transitional milk. The YI study addressed these gaps by providing longitudinal evidence on preterm infant health, offering dynamic data and associations across developmental stages. All samples and data have been meticulously recorded following standardized workflows and formats. Currently, analyses of lipids, proteins, amino acids and HMOs in breast milk, as well as microbiome and metabolome analyses of breast milk and feces have been completed, with results expected to be available next year.

There are several limitations. Firstly, medical interventions for preterm infants during hospitalization may influence the study purpose. We recorded the illnesses and medications of the mother-infant dyads both during hospitalization and after discharge, allowing for the application of complex statistical models to control for potential confounders and mitigate their effects. Second, the specificity of the study subjects led to a relatively small sample size, which impacted the generalizability and statistical power of the findings. Nevertheless, the high compliance of this study (>80%) enhances the quality of the data, partially offsetting the limitations associated with sample size. Third, the follow-up duration was relatively short, extending only to 6 months of age, compared to other long-term birth cohort studies such as the Shanghai Birth Cohort (SBC) and the Finnish Health and Early Life Microbiota (HELMi) cohort (49–51). However, the homogeneity of infant food sources during the first 6 months of life facilitates the exploration of the cohort’s primary purposes. Beyond 6 months, the introduction of diverse complementary foods introduces additional confounding factors that may complicate the analysis. Finally, all participants in this study were recruited from Xi’an and Lanzhou, which may limit the representativeness of the findings for preterm mother-infant dyads across broader geographic and demographic contexts.

## 5 Conclusion

This protocol outlines a longitudinal dynamic cohort of the mother-breastmilk-preterm infant triad. Through ultra-early, multi-temporal, multi-sample collection, combined with multi-omics analyses, the YI study will provide an opportunity to explore the dynamic association of breast milk as a complex biological system with gut microecology and health in preterm infants in depth.

## Data Availability

The raw data supporting the conclusions of this article will be made available by the authors, without undue reservation.
